# The Evaluation of Cellulose Acetate Capsules Functionalized for the Removal of Cd(II)

**DOI:** 10.3390/polym15193917

**Published:** 2023-09-28

**Authors:** Irma Pérez-Silva, Gladis D. Canales-Feliciano, José A. Rodríguez, Luis H. Mendoza-Huizar, Salvador Pérez-Estrada, Israel S. Ibarra, M. Elena Páez-Hernández

**Affiliations:** Academic Area of Chemistry, Autonomous University of Hidalgo State, Carr. Pachuca-Tulancingo Km. 4.5, Mineral de la Reforma C.P. 42184, Hidalgo, Mexico

**Keywords:** acetate cellulose, capsules, cadmium, removal, reusability

## Abstract

Cellulose acetate is derived from cellulose and has the characteristics of biodegradability and reusability. So, it has been used for the elimination of toxic compounds capable of producing different diseases, such as cadmium, that result from human and industrial activity. For this reason, capsules functionalized with Cyanex 923 were prepared and characterized by FTIR spectroscopy, Energy Dispersive X-ray Spectroscopy (EDX), and SEM. The functionalized capsules were used for removing and recovering Cd(II) by modifying variables such as HCl concentration in the extraction medium and carrier content in the capsules, among others. The extraction of cadmium from battery leachates and the three isotherm models, Langmuir, Freundlich, and Dubinin Radushkevich, were also tested to model the cadmium removal process. The results showed a favorable physical sorption with a good capacity for extraction and the possibility of reusing the capsules for up to seven cycles without a decrease in the percentage of cadmium recovery.

## 1. Introduction

Cellulose is a semi-rigid, linear, white polymer in a crystalline or amorphous state. It is an abundant polymer in nature presented in different types of plants, agricultural residues, wood, etc.; some characteristics are chemical resistance, biodegradability, durability, biocompatibility, thermal stability, and renewability [1,2]. Despite its advantages, cellulose has a low solubility in organic compounds, limiting its application. For this reason, cellulose has been modified by different processes to obtain some derivatives, such as cellulose sulfate, cellulose nitrate, methyl and ethyl cellulose, carboxymethyl cellulose, and cellulose acetate [3].

Cellulose acetate (CA) produces the acetylation of cellulose with acetic anhydride and acetic acid in the presence of sulfuric acid as a catalyst. It is a non-toxic, reusable, biodegradable, mechanical strength, and hydrophobic polymer [4,5]. Some uses of cellulose acetate are plastics production, fibers, textiles, and analytical applications such as membrane filters employed for preconcentration and sorbents [6]. For example, CA has been employed with graphene oxide [7] and sulfonated graphene oxide in beds [8], chitosan [9], and lemongrass [10] for the elimination of dyes such as crystal violet and methylene blue. Additionally, membranes [11,12], nanofibers impregnated with hydroxyapatite [13], dithizone nanosponges [14], and microbeads with layered double hydroxides [15] have been used for the adsorption of heavy metals such as lead, chromium, and cadmium. 

Cadmium, a highly toxic metal, has been associated with osteoporosis, renal lesions, cardiovascular disease, and, in some cases, cancer. Because of this, the International Agency for Research on Cancer (IARC) has classified cadmium as a human carcinogen (group I) [16], while the US Environmental Protection Agency (EPA) considers Cd as one of the 13 metals from the priority pollutant list [17].

Cadmium is used for the synthesis of pigments, plastic stabilizers, metal coatings, alloys, electronics, and mainly in the manufacturing of batteries [18]. The use of these types of batteries (in open or closed configuration) has increased due to their versatility, low maintenance, long life, and low cost. For this reason, recycling this type of material is necessary to prevent environmental damage and economic losses [19]. However, some processes for cadmium spent batteries recovery demand high energy consumption. For example, pyrometallurgical processes require using a closed furnace, where metal distillation happens at temperatures between 800 and 900 °C, or a vacuum distillation furnace [20]. Other methods employed for the removal of cadmium from wastewater consist of mechanic and physic separation in acid or elementary solutions followed by ion exchange, solvent extraction (generally organophosphorus such as Cyanex 272, Cyanex 923, D_2_EHPA, TBP), adsorption (activated carbon), precipitation (with sulfates or sodium hydroxide), electrodeposition (complement of other techniques) [21], silica nanoparticles with hydrophilic (OH) or hydrophobic groups (CH_3_) [22], and magnetite or maghemite nanoparticles [23]. Furthermore, for this purpose, some materials based on cellulose have been developed. Because of that, the cellulose has been modified physically by sizes and forms or chemically by functional groups to increase adsorption capacity [24]. The cellulose has been physically (sizes and form) or chemically (functional groups) modified to increase adsorption capacity [24]. A chemical change occurs via substituting hydroxyl groups for other groups by etherification, oxidation, silanization, esterification, cross-link, and graft copolymerization [25]. For instance, cellulose has cheating properties for introducing -C=S, -NH_2_, and -NH groups [26] and for using dibenzo-18-crown 6 [27].

According to the above, easy and environmentally friendly methods for the recovery of cadmium from the acid leachates of the waste that permit the reduction of contamination are necessary. As a result, in this review, functionalized capsules were developed using cellulose acetate and Cyanex 923 as extractant agents [28,29]. These capsules have served as metal extractants due to their large specific interfacial area, high selectivity, and stability. Moreover, these capsules are easy to prepare (only a few seconds are required to form them via phase inversion), with minimal use of organic solvent [30]. The influence of different parameters over the cadmium extraction was evaluated to obtain cellulose acetate capsules functionalized with Cyanex 923, which could become a viable option for the recovery of Cd(II) from aqueous and leached battery solutions with good performance and stability, allowing the extraction of cadmium even from acidic media and with the advantage of using the extracted agent.

## 2. Materials and Methods

### 2.1. Materials

Organic reagents, dimethylformamide (DMF, St. Louis, MO, USA), cellulose acetate (CA) (Mn ~50,000, 39.7 wt.% acetyl, Milwaukee, WI, USA), and hydrochloric acid purchased from Sigma-Aldrich (A.C.S grade, St. Louis, MO, USA); ethanol was purchased from J.T. Baker (ACS, Xalostoc, Edo. Mex, Mexico). The ion carrier trialkyl-phosphine oxides (Cyanex 923) were obtained from Cytec Industries Inc. (Mexico), and Cd(NO_3_)_2_•4H_2_O was obtained from Alyt. All aqueous solutions were prepared in Milli Q water (18.2 MΩ cm resistance, Millipore, Bedford, MA, USA).

### 2.2. Preparation of Functionalized Capsules (FC)

Capsules were prepared during the phase inversion precipitation technique caused by an 18% wt/v CA solution in DMF. Then, different proportions of Cyanex 923 (Cy) (extractant) were added to the previous solution, and the mixture was stirred for 15 min at room temperature. Later, the CA-Cy-DMF solution was placed in a syringe with a needle (o.d. 0.36 mm) and added dropwise into an ethanol–water solution (3:10). In this step, the Cyanex dripped into the non-solvent solution and was trapped in the cellulose acetate due to its low affinity for the aqueous medium. The obtained functionalized capsules FC were washed with deionized water several times and left separately, so any surplus water was drained away overnight [30,31].

### 2.3. Cd(II) Extraction and Recovery Experiments

Different amounts of FC were mixed with a Cd(II) solution of 10 mL and an initial concentration of 10 mg L^−1^ in HCl. The total of Cd(II) extracted (q) was calculated according to the following Equation (1):

(1) $$q=\frac{\left(C_{0}-\;C_{\mathrm{AE}}\right)\times \;V}{112.41\;\times m_{\mathrm{FC}}}$$

q is the cadmium extracted amount (mmol Kg^−1^), C_0_ is the Cd(II) concentration (mg L^−1^) in the initial solution, C_AE_ is the Cd(II) concentration (mg L^−1^) in the solution after the extraction step, V is the aqueous solution volume (L), 112.41 is cadmium atomic weight, and m_FC_ is the amount of functionalized capsules (Kg).

The percentage of extracted Cd(II) (%E_Cd(II)_) was calculated according to the following Equation (2):

(2) $${\%E}_{\mathrm{Cd}(\mathrm{II})}=\left(1-\frac{C_{\mathrm{AE}}}{C_{0}}\right)\times 100$$

C_AE_ is the cadmium concentration in the solution after the extraction step (mg L^−1^), and C_0_ is the initial concentration of Cd(II) (mg L^−1^). The operating variables studied were the mass of FC, the carrier concentration in the casting solution for FC preparation, contact time, the concentration of Cd (II), and cadmium recovery from battery-leaching samples.

For the cadmium recovery studies, FC from the extraction step (loaded with cadmium) was shaken with 10 mL of H_2_SO_4_ 0.1 mol L^−1^ for 0.5 h. The percentage of Cd(II) recovery (%R_Cd(II)_) was calculated according to Equation (3):

(3) $${\%R}_{Cd(II)}=\frac{C_{RS}}{C_{0}-C_{AE}}\times 100$$

C_RS_ is the concentration of cadmium (mg L^−1^) in the sulphuric acid solution after the recovery process, and the other variables have the meaning described before.

All the experiments for the extraction and recovery were accomplished in triplicate at room temperature; Cd(II) concentration in the aqueous solutions was quantified using flame atomic absorption spectrophotometry (AAS) (SpectrAA 880, Varian^®^).

### 2.4. Battery-Leaching Process

Ni–Cd batteries were bought in a local market and dismantled to obtain the cell components separately. One gram of the rolled electrodes was in contact with a mixture of 2 mL of 32 wt% of H_2_SO_4_, 6 mL of deionized water, and 1 mL of H_2_O_2_ 30 vol% and heated for 2 h at 70 °C. The resulting solution was filtered and transferred to a 500 mL volumetric flask, completing the volume with deionized water [32].

## 3. Results

### 3.1. Evaluation of the Amount of Extractant

**Figure 1:**
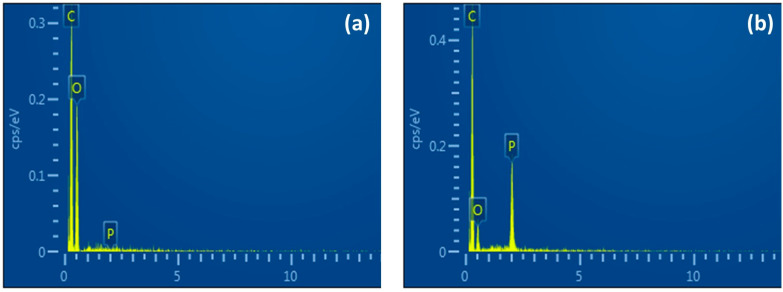
EDX spectrum of (**a**) capsules without Cyanex 923 and (**b**) capsules functionalized with 15.5% of Cyanex 923.

**Figure 2:**
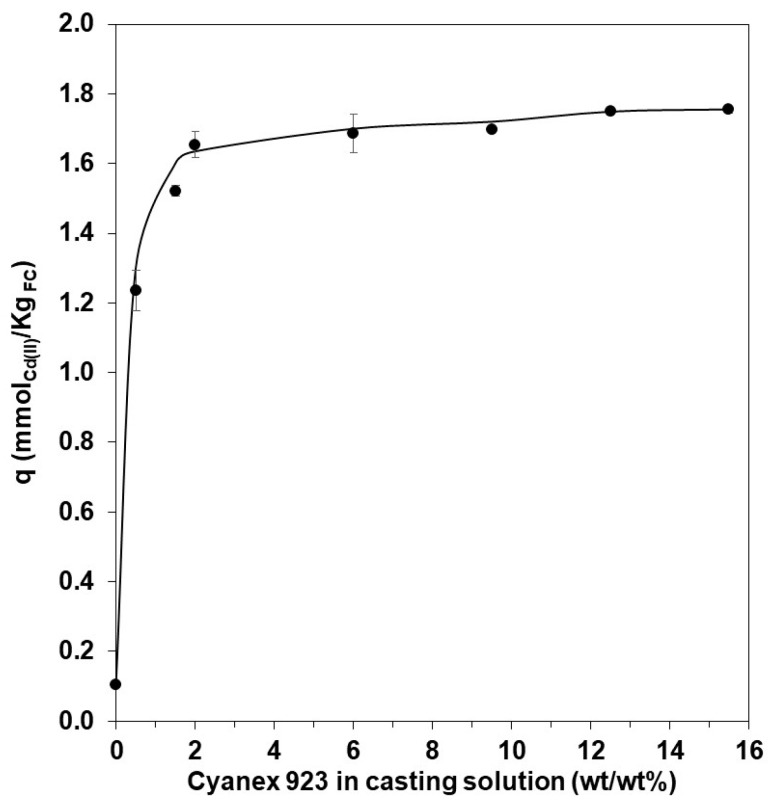
Effect of variation of the concentration of Cyanex 923 used for obtaining the capsules (FC). Experimental conditions: 10 mg L^−1^ of Cd(II) in aqueous solution in 2 mol L^−1^ of HCl; 0.5 g of FC. Values obtained after 2 h were counted from the start of the experiment.

Functionalized capsules (FC) were prepared by adding Cyanex 923 (extractant) to a cellulose acetate/dimethylformamide solution (casting solution). The amount of extractant added to the solution varied from 0 to 15.5 wt/vol%, being 0% for capsules without Cyanex. The resulting capsules, prepared by phase inversion precipitation technique after dropping the above solution into an ethanol–water mixture, were analyzed by Energy Dispersive X-ray Spectroscopy (EDX) in a Scanning Electron Microscopy, JEOL JSM-5600LV model to confirm the presence of Cyanex 923 (Figure 1), and to estimate the phosphorus atomic percentage. The results presented in Table 1 show an increase in the percentage of phosphorus in capsules as Cyanex content increased in the casting solution. This increase in carrier content in FCs is also reflected in a gradual growth in cadmium extracted (Figure 2) when capsules are used to extract Cd from aqueous solutions.

Between these two variables, a directly proportional relationship was demonstrated [33]. Although with higher percentages of Cyanex, better results were obtained, the 1.5% value for future experiments observed the effect that other variables could have on the extraction of cadmium. Nonetheless, it is possible to achieve an extraction close to 100% of cadmium by increasing the content of Cyanex in the casting solution.

**Figure 3:**
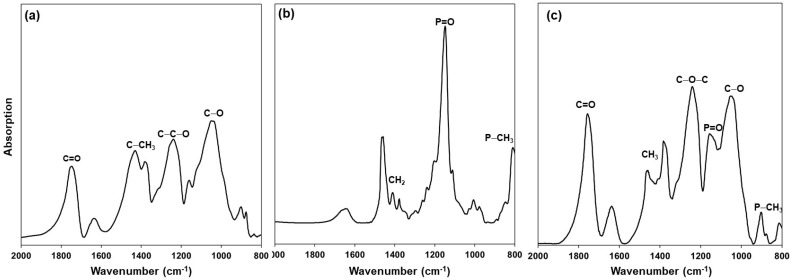
FTIR spectra. (**a**) Cellulose acetate (CA); (**b**) Cyanex 923 extractant agent; (**c**) functionalized capsules (FC).

The presence of extractant in cellulose acetate capsules was determined by IR spectroscopy with a Perkin Elmer System 2000 with Fourier transform (Waltham, MA, USA). The CA spectrum (Figure 3a) shows a band at 1045 cm^−1^ of C-O stretching, a band at 1246 cm^−1^ corresponding to asymmetric stretching of the acetate C-C-O bond, a band at 1370 cm^−1^ for the C-CH_3_ group, and a similar band to C=O bond at 1757 cm^−1^ [34,35]. In the case of Cyanex 923 (Figure 3b), 810 cm^−1^ and 1148 cm^1^ characteristic bands of P-CH_3_ stretching vibration and P=O in each group are identified [36,37]. These same bands and the characteristics of CA and Cyanex 923 are presented in the spectrum of Figure 3c, demonstrating that the extractant immobilized into the FC.

### 3.2. Variation of the Number of Capsules Used during Extraction Experiments

The relationship between the mass of FC and the cadmium extraction percentage was examined (Table 2). As expected, increasing the mass of FC provides a greater contact growing area in the extraction percentage [38]. With these results, a plot of the logarithm of Cyanex 923 content (phosphorus atomic percentage obtained by EDS analysis) versus the logarithm of distribution ratio, D (ratio of the total amount of cadmium in the FC phase to its total amount in the aqueous phase), gives a slope value around one (0.8575) (R^2^ = 0.9716). It indicates a 1:1 stoichiometric ratio between cadmium and the Cyanex extractant.

### 3.3. Evaluation of the Effect of Chlorhydric Medium on Cadmium Extraction

**Figure 4:**
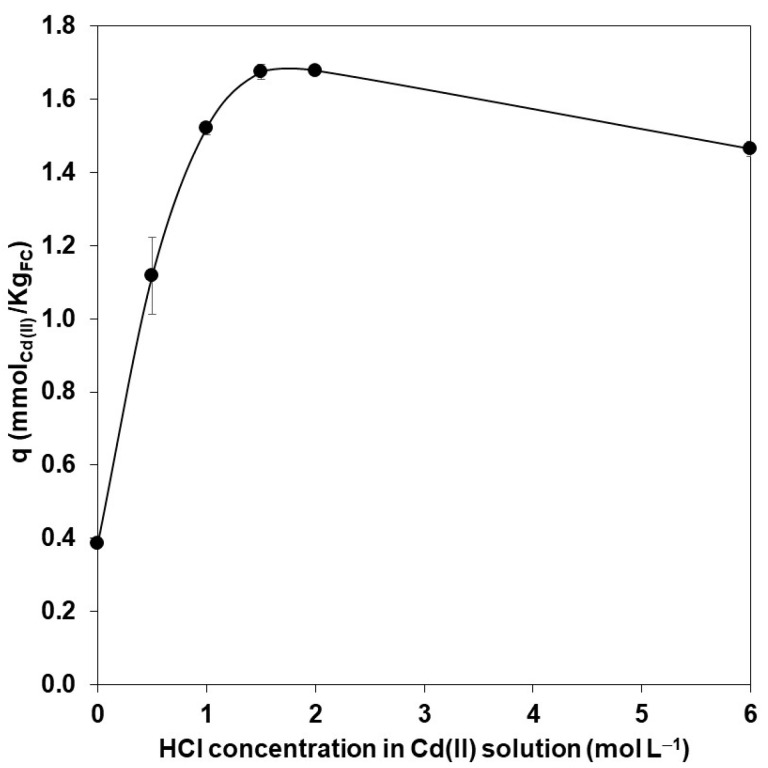
Effect of variation of the HCl concentration in the cadmium extraction. Experimental conditions: 10 mg L^−1^ of Cd(II) in aqueous solution of HCl; 0.5 g of FC prepared with 1.5% of Cyanex 923. Values obtained after 2 h were counted from the start of the experiment.

The extraction of a cation such as Cd(II) with Cyanex depends on neutralizing its charge with an adequate counter ion. Thus, hydrochloric acid was added to cadmium solutions to allow the formation of chlorocomplexes neutralized by the presence of protons. The amount of cadmium extracted in Figure 4 shows the increase in HCl concentration due to the formation of neutral species such as CdCl_2_, HCdCl_3_, and H_2_CdCl_4_ [29] extracted by the solvation mechanism. Nevertheless, at higher HCl concentrations, the mineral acid can also be extracted, competing with cadmium and decreasing the percentage of metal adsorbed [39].

According to the Fraction Diagram (f_Cd(II)_ vs. logCl^−^) obtained through the MEDUSA program [40] at −0.7 pH value and 8.9 × 10^−5^ mol_Cd(II)_ L^−1^ concentration, the predominant cadmium chemical species is CdCl_2_. With this information and knowing the stoichiometric relationship between Cd and Cyanex (previous section), the following reaction can be considered representative of the extraction process, where the upper line refers to the chemical species in the FC organic phase (Equation (4)):

(4) $${\mathrm{CdCl}}_{2}+\bar{\mathrm{Cy}}↔\bar{{\mathrm{CyCdCl}}_{2}}$$

### 3.4. Extraction Isotherms

Extraction isotherms describe the change between the initial and final amount of metal presented in a solution due to the FC at equilibrium [41]. To perform the extraction isotherms, the time the system needed to reach equilibrium was first determined (Table 3). According to this, the selected time was 5 h; although it is a prolonged time of agitation, it did not harm the extraction of Cd(II).

**Figure 5:**
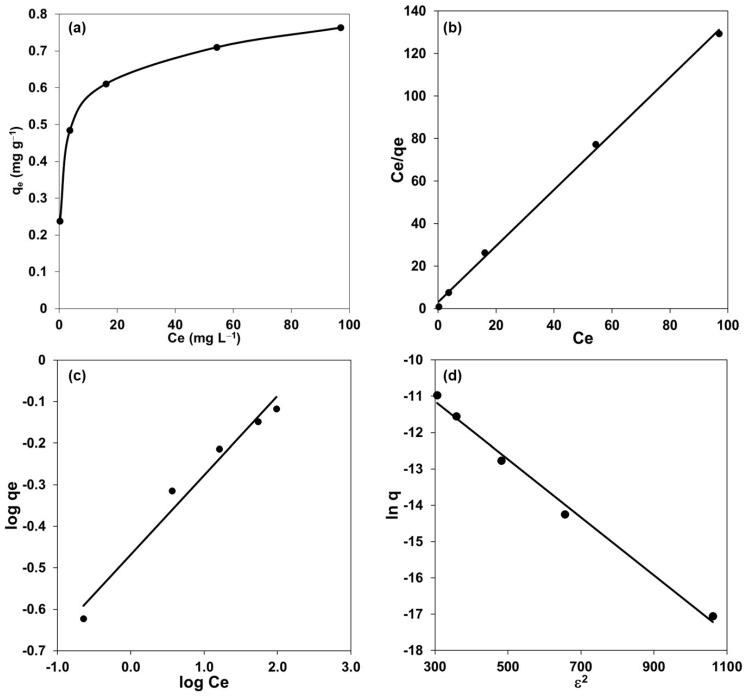
Isotherms curves: (**a**) experimental data and linearized forms of (**b**) Langmuir isotherm, (**c**) Freundlich isotherm, and (**d**) Dubinin Radushkevich isotherm. Experimental conditions: different amounts of Cd(II) in aqueous solution of 1.5 mol L^−1^ HCl; 0.5 g of FC prepared to 1.5% of Cyanex 923.

The experimental equilibrium data were fitted to three isotherm models: Langmuir, Freundlich, and Dubinin Radushkevich (D-R) [42,43]; this is in a range of cadmium concentrations from 10 to 150 mg L^−1^. On one side, the experimental results and linearized forms of different models are shown in Figure 5. On the other side, Table 4 predicted a monolayer coverage of the adsorbate on the outer surface of the FC with favorable sorption (0.01 < R_L_ < 0.15) [44].

Referring to the D-R isotherm allowed us to identify the nature of the interaction between Cd(II) and the extractant as a physical sorption [45], which agrees with the characteristics of a solvation process. 

Although the cadmium extraction capacity (Qo) obtained for FC is lower than those reported for other types of polymeric materials, such as solvent-impregnated resins (SIR) and alginate capsules (Table 5), in this work, the FC has a smaller amount of extractant (22.5 mg_Cyanex_ g_FC_^−1^). Moreover, the amount of Cyanex used in this work is enough to reach extraction percentages above 95%, as well as those reported in papers with similar cadmium concentrations.

Notwithstanding the above, it is relevant to highlight that, to observe the effect of different variables on the percentage of cadmium extraction, the maximum concentration of Cyanex in the casting solution was not used, so increasing the FC adsorption capacity is achievable, as is the extraction percentage, by modifying this variable during its preparation.

### 3.5. Kinetic Studies

**Figure 6:**
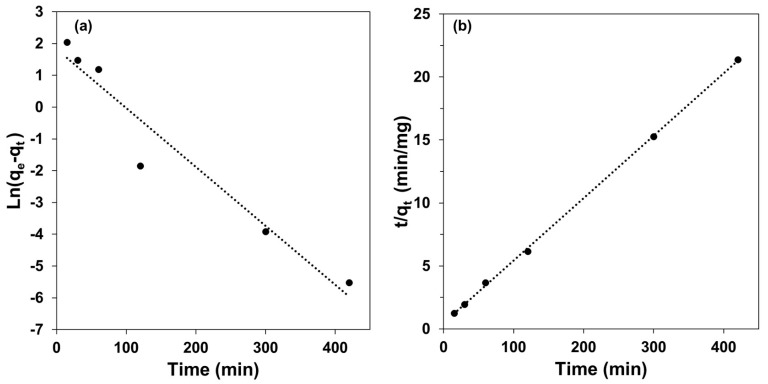
Curves of the two kinetic models evaluated: (**a**) pseudo-first-order and (**b**) pseudo-second-order. Experimental conditions: 10 mg L^−1^ of Cd(II) in an aqueous solution of 1.5 mol L^−1^ HCl; 0.5 g of FC prepared with 1.5% of Cyanex 923.

Kinetic studies describe the amount of analyte in the capsules concerning the residence time, considering that the sorption depends on the characteristics of the sorbent [51]. For this reason, pseudo-first-order and pseudo-second-order kinetic models were evaluated [52]. Figure 6 and Table 6 show the curves and parameters of the two kinetic models considered. The experimental data provided the best correlation with the pseudo-second-order model, which indicates that the sorption mechanism depends on the amount of Cd(II) near active sites, showing that chemisorption is the rate-determining step of the sorption [53]. 

### 3.6. FC Reusability Study

**Figure 7:**
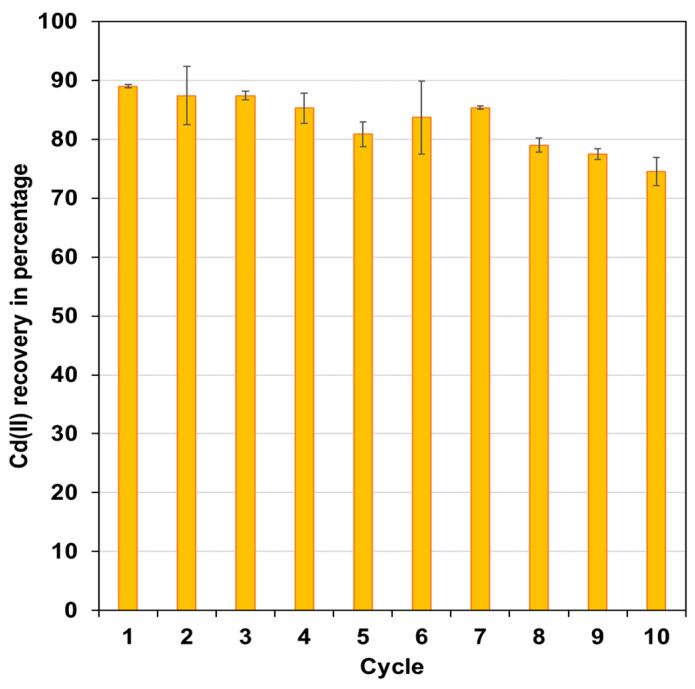
Percentage of cadmium recovery after re-extraction process. Experimental conditions: 10 mg L^−1^ of Cd(II) in aqueous solution of 1.5 mol L^−1^ of HCl; 0.5 g of FC prepared with 1.5% of Cyanex 923. Values obtained after 2 h were counted from the start of the experiment for extraction and 0.5 h for recovery.

Sulphuric acid is an effective re-extracting agent because it can form more stable complexes with cadmium in aqueous medium than chloride [49]. Thus, H_2_SO_4_ was used to recover cadmium from the functionalized capsules (10 mL of H_2_SO_4_ 0.1 mol L^−1^ in each experiment). Subsequently, the cadmium-free FCs were used again for extracting Cd(II), completing a new cycle. With these results, it was possible to evaluate the stability and durability of the FC, inferring a loss of its extraction capacity after the seventh cycle (Figure 7).

**Figure 8:**
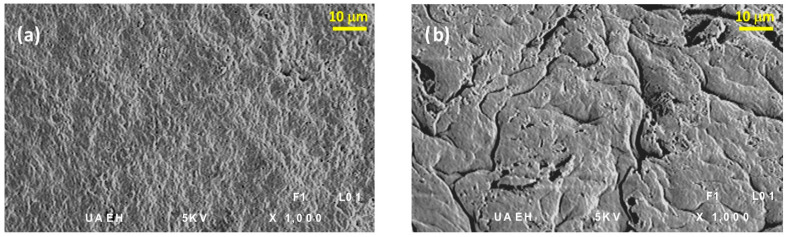
Scanning electron micrograph (1000×) of different FC: (**a**) cellulose acetate with 1.5% Cyanex 923 and (**b**) cellulose acetate with 1.5% Cyanex 923 after 10 extraction–recovery cycles.

At the beginning of the experiments, the photomicrographs of FC were obtained to support this observation (Figure 8, on the left). On the contrary, Figure 8, on the right, was obtained after ten cycles of use. Unlike when they were freshly prepared, the used FCs show a rough and weathered surface because of structural damage. From the previous results, it might be that the process of efficiency is expressed as cadmium extraction decreases after seven cycles. Moreover, this sorbent material is made of cellulose acetate, a biodegradable biopolymer. It represents a phenomenal advantage over other functionalized polymeric materials, such as SIR [47] or polysulfone microcapsules [54], which maintain their extraction performance only for five cycles.

### 3.7. Recovery of Cd(II) from a Real Sample

Cadmium recovery studies from actual samples were carried out with a battery-leaching solution prepared according to the Battery-leaching Process section (see Section 2.4). From this stock solution, a 10 mg L^−1^ solution was prepared and used for the extraction and recovery of Cd(II) with FC. The obtained results (qCd = 2.06 mmol Kg−1, %ExtractionCd = 88.28% ± 0.76%, and %RecoveryCd = 79.59% ± 1.80%) are lower than those obtained with synthetic aqueous solutions. It can be attributed to the presence of nickel that competes to a certain degree with cadmium during the extraction with Cyanex. Although it interferes negatively with the extraction of cadmium, the nickel extraction percentage is less than 8% (7.27 ± 2.63%), indicating a good selectivity of the developed method.

## 4. Conclusions

The recovery of Cd(II) from aqueous HCl solutions with cellulose acetate capsules functionalized with Cyanex 923 was successful. The transfer mechanism is based on physical sorption and obeys a 1:1 stoichiometry between carrier and metal.

The best conditions for the extraction of cadmium were achieved using a 1.5 mol L^−1^ HCl solution during a stirring time of 2 h with 0.5 g of functionalized capsules prepared with 1.5% Cyanex 923. An 88% percentage of cadmium recovery from cadmium-loaded FC using 10 mL of a 0.1 mol L^−1^ H_2_SO_4_ solution during a stirring time of 0.5 h. Thereby, functionalized capsules can be reused up to seven times without significant changes in their cadmium extraction capacity.

The above demonstrates that it is possible to use a biopolymer such as cellulose acetate for the recovery of cadmium from battery leachates with good selectivity through a simple and environmentally friendly process.

## Figures and Tables

**Table 1:** Phosphorus atomic percentage in Cyanex functionalized capsules.

Cyanex 923 in Casting Solution (wt/vol%)	Phosphorous Atomic Percentage (%)
0.00	0.00
0.50	0.08
1.50	0.11
2.00	0.19
6.00	1.75
9.50	2.24
12.50	2.42
15.50	2.67

**Table 2:** Effect of variation of the mass of FC used in the cadmium extraction ^1^.

FC Mass (g)	Cadmium Extraction in Percentage ^2^ (%)
0.05	18.63 (3.56)
0.20	59.10 (3.84)
0.30	79.07 (1.19)
0.40	79.51 (6.13)
0.50	85.50 (1.18)
1.00	96.49 (0.03)

^1^ Experimental conditions: 10 mg L^−1^ of Cd(II) in aqueous solution in 2 mol L^−1^ of HCl; FC prepared with 1.5% of Cyanex 923. Values were obtained after 2 h counted from the start of the experiment. ^2^ Percentage of relative standard deviation in parenthesis.

**Table 3:** Effect of the contact time in the extraction percentage ^1^.

Contact Time (h)	Cadmium Extraction in Percentage ^2^ (%)
0.25	59.58 (2.10)
0.50	76.41 (2.83)
1.00	81.76 (3.94)
2.00	97.46 (0.67)
5.00	98.15 (0.17)
7.00	98.23 (0.68)

^1^ Experimental conditions: 10 mg L^−1^ of Cd(II) in aqueous solution 1.5 mol L^−1^ of HCl; 0.5 g of FC prepared with 1.5% of Cyanex 923. ^2^ Percentage of relative standard deviation in parenthesis.

**Table 4:** Dubinin Radushkevich, Freundlich, and Langmuir constants for sorption of Cd(II) onto FC.

Model	Estimated Isotherm Parameters ^1^	
Langmuir	Q_o_ (mg g^−1^)	0.77
$\frac{C_{e}}{q_{e}}=\frac{1}{q_{max}K_{L}}+\frac{C_{e}}{q_{max}}$	K_L_ (L mg^−1^)	0.52
	R^2^	0.998
Freundlich	K_F_ (L g^−1^)	0.34
${logq}_{e}=logK_{f}+\frac{1}{n}logC_{e}$	n_o_	5.24
	R^2^	0.972
Dubinin Radushkevich	β (mol^2^ J^−2^)	0.008
$Inq_{e}=Inq_{max}-\beta \varepsilon^{2}$	ε (KJ mol^−1^)	7.91
	R^2^	0.993

^1^ Maximum extraction capacity, Q_o_ (mg g^−1^); Langmuir constant, K_L_ (L mg^−1^); Freundlich constant, K_F_ (L g^−1^); free strength of sorption, n_o_, Dubinin Radushkevich constant, β (mol^2^ J^−2^); Polanyi potential, ε (KJ mol^−1^); coefficient of correlation, R^2^.

**Table 5:** Efficiency in the cadmium extraction obtained with impregnated materials with different extracting agents.

Q_o Cd(II)_ (mg_Cd(II)_ g^−1^)	q_extractant_ ^4^ (mg_extractant_/g_material_)	Extractant	Reference
1.56	77 (XAD2) ^1^	Cyanex 301	[46]
11.5	138 (XAD7)^1^	Cyanex 301	
4.89	99	Cyphos^®^ IL-101 ^2^	[47]
36.57	416	Cyphos^®^ IL-101 ^2^	
13.54	271	DEHPA ^3^	[48]
41.06	500	DEHPA ^3^	
13.1	366	Cyanex 921	[49]
17.5	528	Cyanex 921	
15–42	94–290	Cyanex 301	[50]
14–34	204–481	Cyanex 302	

^1^ Amberlite XAD-2 and XAD-7; ^2^ phosphonium salt (tetradecyl(trihexyl)phosphonium chloride; ^3^ Di(2-ethylhexyl) phosphoric acid; ^4^ q_extractant_ = amount of extractant.

**Table 6:** Kinetic parameters for sorption of Cd(II) onto FC.

Model	Estimated Kinetic Parameters ^1^	
Pseudo-first-order	q_e_ (mg g^−1^)	6.203
$\ln\left(q_{e}-q_{t}\right)=lnq_{e}-K_{1}t$	K_1_ (min^−1^)	−4.40 × 10^−5^
	R^2^	0.943
Pseudo-second-order	q_e_ (mg g^−1^)	20.20
$\frac{1}{q_{t}}=\frac{1}{K_{2}q_{e}^{2}}+\frac{t}{q_{e}}$	K_2_ (g mg^−1^ min^−1^)	0.005
	R^2^	0.999

^1^ Cadmium extracted at equilibrium, q_e_; constant of the pseudo-first-order, K_1_ (min^−1^); constant of the pseudo-second-order, K_2_.

## Data Availability

Not applicable.
